# The Double-Edged Sword Effect of Abusive Supervision on Subordinates’ Innovative Behavior

**DOI:** 10.3389/fpsyg.2019.00066

**Published:** 2019-01-25

**Authors:** Jinqiang Zhu, Bainan Zhang

**Affiliations:** ^1^School of Management, Minzu University of China, Beijing, China; ^2^School of Labor and Human Resources, Renmin University of China, Beijing, China

**Keywords:** abusive supervision, psychological safety, challenge-related stress, innovative behavior, structural equation model – SEM

## Abstract

Existing studies on the relationship between abusive supervision and innovative behavior do not present a united picture. Drawing up the antecedent-benefit-cost framework and social cognitive theory, we tried to explain the contradictory relationships between them based on the mediating mechanism. Results showed that abusive supervision discouraged subordinates’ innovative behavior through reducing subordinates’ psychological safety but promoted subordinates’ innovative behavior through enhancing challenge-related stress.

## Introduction

The crucial role of innovation in the survival of organizations stimulates continuing interest among scholars and practitioners alike (Scott and Bruce, 1994). Existing studies showed that leaders play a crucial role in facilitating subordinates’ innovative behavior (e.g., Liu D. et al., 2012; Gu et al., 2017). Therefore, studies about leadership’s impact on subordinates’ innovative behavior have been attracting much attention among scholars. Recently, organizational researches have been increasingly focused on the “dark side of leadership” (e.g., Liu D. et al., 2012; Lee et al., 2013). An important reason for such interest is due to the increasing frequency of abusive supervision behaviors in the workplace (Lee et al., 2013), and their considerable impact on organizational and individual outcomes (Tepper, 2007). In particular, abusive behaviors have been shown to be relatively more frequent in China (Liu W. et al., 2012) due to the culture of high power distance and have been considered to be an important factor that may impact subordinates’ innovative behavior (Gu et al., 2017). The focus of prior researches about abusive supervision’s effect on innovative behavior has been predominantly centered on its negative side (Liu D. et al., 2012; Liu W. et al., 2012). However, as the saying goes in China, “spare the rod and spoil the child." Some scholars have begun to argue that abusive supervision is necessary for management practice (Oh and Farh, 2017; Zhang and Liu, 2018). For example, Oh and Farh (2017) proposed that anger and fear from abusive supervision may positively relate to promotion-focused work efforts, and these efforts might hold values for the organization. Zhang and Liu (2018) argued abusive supervision may prevent those negative behaviors from happening again, when it was initiated due to employees’ poor performance, mistakes that could be prevented, and their counter-workplace behaviors. Furthermore, Tepper and Park (2017) argued that abusive supervision could have a positive effect on employee’s behavior through the pathway of performance enhancement. Some recent empirical studies support these argues (Lee et al., 2013; Mitchell et al., 2015; Qin et al., 2017). So, how can these conflicting arguments and empirical findings be explained?

Several scholars tried to explain these conflicting findings from the perspective of context. They tried to find moderating variables that influence the relationship between abusive supervision and innovative behavior (Liu D. et al., 2012; Zhang and Liu, 2018). For example, Zhang and Liu (2018) argued that the negative effects of perceived abusive supervision were weaker for subordinates in the high power distance culture versus Anglo culture because subordinates from the former culture context believe that their supervisors’ directives should be respected. Therefore, these subordinates may show deference and have a higher level of tolerance for unfair treatment from authorities. Recently, several scholars pointed out that the effect of abusive supervision on innovative behavior is a complex mediating process (Liang et al., 2015). Ellis (1980) argued that activating events, such as abusive supervision, are only indirect causes of individual behavior, but individual cognition on activating events is the direct cause of the individual behavior. In other word, activating events affect individual behavior by affecting individual cognition. However, as Liu W. et al. (2012) emphasized, it is vital to empirically test the possible psychological mechanisms that may exist between abusive supervision and innovative behavior. Thus, for achieving an effective use of abusive supervision to influence innovative behavior, one critical aspect that must be addressed is to find the role of those psychological cognitive mechanisms. It turned out that the majority of previous studies only emphasized the single cognitive mechanism, mainly focusing on the negative influence of abusive supervision on innovative behavior (Han et al., 2017). Busse et al. (2016) developed an antecedent-benefit-cost framework and proposed that a dependent variable could be affected by two (or more) mediators with opposite directionalities of influence, which are caused by a common antecedent variable. Accordingly, the term *benefits* is used to denote desirable immediate outcomes, while the term *costs* is used to denote any undesirable immediate outcomes. The total effect of the antecedent on the dependent is the result of two competing indirect effects. The antecedent-benefit-cost framework has been supported by empirical studies (Gu et al., 2017). For example, Gu et al. (2017) found that inclusive leadership had a positive effect on creativity through psychological safety and a negative effect on creativity through follower’s dependency. Drawing up the antecedent-benefit-cost framework, we argue that abusive supervision has opposite directional influences on innovative behavior through two different mediating variables.

**FIGURE 1:**
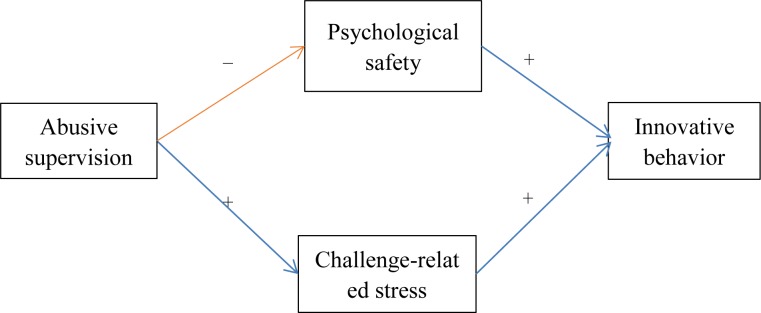
Proposed four-factor theoretical model (−, discourages/reduces; +, promotes/enhances).

Using this argument, the current paper aims to identify the underlying mediating mechanisms of abusive supervision which may affect innovative behavior in opposite directions. The social cognitive theory tells us that external factors affect individual behavior through individual psychological cognition (Bandura, 2001). Vogel and Mitchell (2015) identified two theoretical perspectives (self-defense view and self-presentational view) to explore the underlying mechanisms of abusive supervision affecting individual behavior. The self-defense view suggests that abused subordinates become motivated to protect their sense of self to avoid further abusive treatment. The self-presentational view indicates that abusive supervision motivates efforts from victims to signal that they are a valuable member of the social group. By integrating social cognitive theory and findings of previous studies, we argue here that abusive supervision discourages subordinates’ innovative behavior through self-defense mechanism (such as reducing subordinates’ psychological safety) but promotes subordinates’ innovative behavior through self-presentational mechanism (such as enhancing subordinates’ challenge-related stress). The theoretical model is depicted in Figure 1.

## Theory and Hypotheses

### Self-Defense Mechanism: The Mediating Effect of Psychological Safety

Social cognitive theory teaches us that most of the external factors affect behaviors through cognitive processes rather than directly (Bandura, 2001). Building on this theory, a particular leadership style is an important external factor perceived by employees, and psychological safety acts an underlying cognitive process that links leadership style to employee’s behavior. Psychological safety is defined as the subjective perception that “*feeling able to show and employ one’s self without fear of negative consequences to self-image, status, or career*” (Kahn, 1990, p. 708). Abusive supervision refers to “*subordinates’ perceptions of the extent to which supervisors engage in the sustained display of hostile verbal and non-verbal behaviors, excluding physical contact”* (Tepper, 2000, p. 178). For example, speaking rudely to subordinates to elicit desired task performance and public criticism is a kind of abusive supervision behavior. This definition characterizes abusive supervision as a subjective assessment.

In the social cognitive theory, subordinates will feel threatened when they are abused by supervisor (Tepper, 2000), thereby reducing subordinates’ psychological safety. Abusive supervision will also make subordinates feel supervisor hostile and inaccessible to the supervisor, thus increasing subordinates’ negative cognition on the supervisor and reducing subordinates’ psychological safety. When the supervisor mocks, humiliates or insults subordinates, such behaviors will inevitably undermine the relationship between the supervisor and subordinates, thereby reducing subordinates’ psychological safety. In addition, abusive supervision also reduces subordinates’ trust in the organization (Tepper, 2000), which is harmful to subordinates’ psychological safety perception (Li and Yan, 2007). Existing empirical research showed that violence exposure significantly lowers subordinates’ psychological safety (Fang et al., 2014). Thus, we argue that abusive supervision negatively influences subordinates’ psychological safety.

Innovation refers to the production or adoption of useful ideas and ideas implementation, including the generation of ideas and solutions, an individual seeking sponsorship for an idea, an individual completing the idea (Scott and Bruce, 1994). Innovation, by nature, introduces novelty and increases uncertainty (Carmeli et al., 2010). Due to its novelty, innovation is more likely to fail. Innovation tends to be a risky endeavor, therefore, it is not surprising that an individual will avoid innovative behavior in that he or she is afraid of being punished for failing the innovation. Thus, subordinates will assess the unfavorable factors (such as abusive supervision) of innovation and their possible negative consequences of failure in innovation before engaging in the innovative behavior. West and Richter (2008) found that when facing psychological threats and feeling psychologically unsafe, employees are more likely to develop defensive orientation and are less likely to display innovative behaviors at work. Existing empirical research showed that psychological safety could improve employees’ voice behavior, such as speaking up and keeping the willingness to question and providing suggestions for change (Liang, 2014). When employees are comfortable to speak up, they are more likely to make innovative ideas known (Carmeli et al., 2010).

Above all, abusive supervision makes subordinates’ feel psychologically unsafe to engage in the innovative behavior. From the perspective of self-defense, this unsafe feeling will make subordinates less likely to perform the innovative behavior, because they need to avoid further abusive treatment due to the failure of innovation. Research showed that psychological safety is an underlying cognitive mechanism that links leadership and employees behavior (Carmeli et al., 2010; Hirak et al., 2012). For example, psychological safety mediated the relationship between abusive supervision and voice behavior (Wu et al., 2012). Hence, the following hypothesis is suggested:

Hypothesis 1: Abusive supervision has a negative effect on innovative behavior by reducing subordinates’ psychological safety.

### Self-Presentational Mechanism: The Mediating Effect of Challenge-Related Stress

Work stress is the event or situation that requires employees to have certain adaptive responses (Beehr and Newman, 1978; Chou et al., 2014). The negative consequences of work stress have dominated theories and studies for a long time (Chou et al., 2014). For example, it is known that work stress harms organizational and individual performance (Amabile et al., 1990; Byron et al., 2010). However, there are studies showed that work stress has a positive impact on job satisfaction (Chou et al., 2014), job performance (Hon and Chan, 2013), and creativity (Qing and Zhang, 2014). It needs to be noted that inconsistent results from previous investigations may have resulted from different types of work stress studied. Selye (1955) suggested that stress is essential to human evolution and growth, and does not necessarily harm people. He divided stress into positive stress and negative stress, and indicated that a person who can adapt to positive stress generates positive feelings and outcomes (Selye, 1976, 1982). Based on previous studies, Cavanaugh et al. (2000) divided work stress into challenge-related stress and hindrance-related stress. The former refers to stress related to positive work outcomes and creates challenge and feelings of fulfillment or achievement. Examples of this category of work stress include job overload, time pressures, and high levels of responsibility. The latter refers to stress related to negative work outcomes and hinders an individual’s ability to achieve valued goals. Examples of this category of work stress include organizational politics, red tape, and concerns about job security (Cavanaugh et al., 2000).

In the workplace, leadership behavior is an important event that causes employee stress and is an essential source of stress for employees (Li et al., 2012). Public criticism, ridicule, or insult from an abuser may be perceived as a social assessment threat by subordinates (Tepper, 2000). Those events related to threats are considered as stress events by employees and trigger psychological stress (Lazarus and Folkman, 1984; Folkman et al., 1986). Abusive behaviors from leaders, such as telling me ‘I’m incompetent’ (Mitchell and Ambrose, 2007), make subordinates think that they did bad work, and that leaders expect high job performance (Lv, 2013), which may cause stress in subordinates. Based on the self-presentational view, in order to meet the expectations of the supervisor and demonstrate an employee’s value to the organization, subordinates will be motivated to engage in innovative behavior that is significant to the organization. Existing empirical research showed that abusive supervision could inspire subordinates’ job passion and improve organizational performance (Lv, 2013). Hence, the following hypothesis is suggested:

Hypothesis 2: Abusive supervision has a positive effect on innovative behavior through motivating subordinates’ challenge-related stress.

## Materials and Methods

### Sample and Procedure

We collected data from full-time employees in mainland China (including Beijing and Shanghai) at two points in time. The data were collected in China via an online platform named “sojump.com.” During data collection, several researchers who have been trained in conducting surveys visited with the participants. The researchers explained to all participants the aims of the research project and the survey procedure, and then sent them the link to the questionnaire website. A non-probabilistic sampling method, namely convenience sampling, was used in our data collection. We compensated each participant with ten Chinese yuan (about 1.545 USD) for their participation and time.

Participants reported on demographics and abusive supervision at time 1, and about 1 month later (at time 2), they reported on their psychological safety, challenge-related stress and innovative behavior. The two-wave data were matched by participants’ email. A total of 402 individuals consented to participate and completed the Time 1 survey. Our analyses included a total of 253 observations with complete data for our study’s variables across the Time 1 and Time 2 surveys. Results of *t*-tests demonstrated no significant differences on demographics or Time 1 variables (i.e., abusive supervision) existed between the Time 2 responders and non-responders (Dooley and Lindner, 2003). 55.30% of participants were male, 66.40% were married, 60.3% of participants were in non-supervisory positions, and 53.40% had a bachelor’s degree or above. On average, participants’ age was 31.82 years (*SD* = 6.69) and company tenure was 9.19 years (*SD* = 6.80).

### Measures

The survey was conducted in Chinese, employing back translation (Brislin, 1970). An expert in organizational behavior research was asked to check the content of the items and five employed graduate students were asked to complete the questionnaire to check its clarity. In this manner, we ensured that participants could clearly understand all items.

#### Abusive Supervision

Abusive supervision was assessed using Mitchell and Ambrose (2007) five-item short version of Tepper (2000) measure. Subordinates’ perceptions of supervisor’s abuse (e.g., “My boss puts me down in front of others”) were measured on a 6-point scale from 1 (never) to 6 (always). The Cronbach’s alpha score was 0.94.

#### Psychological Safety

Psychological safety was assessed with Li and Yan (2007) five-item measure. The scale’s reliability was not good because of one reverse-worded item, which was tested by the corrected item-total correlation (CITC). The value of CITC of the reverse-worded item was −0.11 less than 0.4, so we dropped the item. The four items exhibited high internal consistency (α = 0.84). A sample item is “There is a threatening environment at work” (1 = strongly disagree, 6 = strongly agree).

#### Challenge-Related Stress

Challenge-related stress was assessed with Cavanaugh et al. (2000) six-item measure. Respondents were asked to rate how much stress each item causes them on a 5-point scale from 1 (no stress at all) to 5 (a great deal of stress). The Cronbach’s alpha score was 0.94.

#### Innovative Behavior

Innovative behavior was assessed with Scott and Bruce (1994) six-item measure. Respondents indicated how often they engaged in each of the behaviors (e.g., “Searches out new technologies, processes, techniques, and/or product ideas”) on a 6-point scale ranging from 1 (never) to 6 (always). The Cronbach’s alpha score was 0.94.

#### Control Variables

We controlled for several relevant factors in order to carry out a conservative test of our hypotheses and to rule out alternative explanations. Prior research has shown that demographic variables such as gender, age, marry, tenure, education and position are likely to be associated with innovative behavior (e.g., Zhang et al., 2014). Therefore, we controlled for these demographic variables in our data analyses. Age and organizational tenure were self-reported in years. Gender and marry were dummy coded, with male coded as 0, female coded as 1, single coded as 0 and married coded as 1. Education was coded as 1 for participants who had finished a middle school education or below, 2 for participants who had finished a high school education, 3 for participants who had an associate’s degree, 4 for participants who had a bachelor’s degree, and 5 for participants who had a postgraduate degree. Position was coded as 1 for the non-supervisory positions, 2 for the middle manager, and 3 for the top manager.

### Data Analysis

We used SPSS 22.0 (SPSS Inc., Chicago, IL, United States) and Mplus 7.0 (Muthén and Muthén, 2010) for data analysis. First, confirmatory factor analyses (CFAs) were conducted to assess the discriminant validity of the key variables and the common method variance (CMV) was examined. Second, a two-step structural equation model (SEM) program was used to evaluate the measurement model, the theoretical model (see Figure 1) and the alternative model (adding the direct path from abusive supervision to innovative behavior based on the theoretical model). Then, the optimal model (Anderson and Gerbing, 1988) could be chosen. Third, the bias-corrected bootstrapping and Bayesian analysis were used to test the mediation because of their high power (Preacher and Hayes, 2008; Miočević et al., 2017).

We analyzed the covariance matrix using the maximum likelihood procedure. Following the recommendations by Hu and Bentler (1999) as well as Cheung and Rensvold (2002), we used multiple fit indices, including the χ^2^/df (relative/normed chi-square), the Comparative Fit Index (CFI), Tucker-Lewis Index (TLI), and the Root Mean Square Error of Approximation (RMSEA). A χ^2^/df ratio of lower than 5 indicates a good fit (Kelloway, 1998). CFI and TLI surpassing 0.90 indicate a good fit (Cheung and Rensvold, 2002). The RMSEA smaller than or equal to 0.05 signals close fit; values between 0.05 and 0.08 indicate a reasonable fit (Hu and Bentler, 1999).

## Results

### Discriminant Validity

Using Mplus, a series of CFAs was conducted to confirm the discriminant validity of the variables. The CFA results indicate that our proposed four-factor model (abusive supervision, psychological safety, challenge-related stress and innovative behavior) yielded a better fit than other constrained models (see Table 1).

**Table 1:** Results of confirmatory factor analyses.

	χ^2^	*df*	χ^2^/*df*	RMSEA	CFI	NNFI	Δχ^2^	Δ*df*
Model 1 (hypothesized four-factor model)	412.38	183	2.25	0.07	0.95	0.94		
Model 2 (psychological safety and challenge-related stress combined)	819.80	186	4.41	0.116	0.85	0.83	407.42^∗∗∗^	3
Model 3 (abusive supervision, psychological safety and challenge-related stress combined)	1873.72	188	9.97	0.188	0.61	0.56	1461.34^∗∗∗^	5
Model 4 (single-factor model)	3118.90	189	16.50	0.248	0.32	0.25	2706.52^∗∗∗^	6
Model 5 (unmeasured latent methods factor model)	302.00	162	1.86	0.058	0.97	0.96	110.38^∗∗∗^	21

*N = 253, ^∗^p < 0.05, ^∗∗^p < 0.01, ^∗∗∗^p < 0.001.*

### Common Method Variance

Common method variance is a potential issue as a result of the self-reporting approach from the same source. To examine CMV, we conducted Harman’s one-factor test by exploratory factor analysis (EFA) using SPSS and CFA using Mplus, which examines whether most of the variance can be accounted for by one factor (i.e., common variance) (Podsakoff et al., 2003). If the first emerging factor accounts for over 50% of the variance in EFA, common method bias is suggested. Moreover, we compared the model fit of the single-factor model with the model fit of the hypothesized model using CFA. If the former is as good as the latter, CMV would be a problem. Harman’s one-factor test was conducted by including all of the items of the four variables in our study. The first emerging factor accounted for 28.53% of the explained variance. Compared with the four-factor model, the fit of the single-factor model was poorer, and the change in chi-square was significant [Δχ^2^(6) = 2706.52, *p* < 0.001] (see Table 1). Therefore, the results suggest that the common method bias is not a significant problem in the current study.

Given that Harman’s single-factor examination is insensitive in examining CMV, we further examined and controlled for an unmeasured latent methods factor, with all the items loaded on this latent methods factor and trait factors they were assumed to measure (Podsakoff et al., 2003). A comparison of the latent methods factor model and the theoretical model indicated a slight change of chi-square value [Δχ^2^(21) = 110.38, *p* < 0.001]. Previous researchers have pointed out that chi-square values are easily impacted by sample size especially when the sample size is larger than 200 (Cheung and Rensvold, 2002). As such, Little (1997) suggests examining the non-normed fit index (NNFI) for model choice. If NNFI change is smaller than 0.05, it indicates that adding the latent methods factor does not significantly improve the theoretical model. Given that the sample size of this current study was 253, we followed Little (1997) advice and found that NNFI increased by 0.02 when the latent methods factor was included. This result showed that adding a latent methods factor did not significantly improve the model and thus CMV did not have a significant impact on variable relationships.

### Descriptive Statistics

The means, standard deviations, and correlation matrices of the key variables are presented in Table 2. As shown, abusive supervision was negatively correlated with psychological safety (*r* = −0.41, *p* < 0.001), positively correlated with challenge-related stress (*r* = 0.22, *p* < 0.01), negatively correlated with innovative behavior (*r* = −0.17, *p* < 0.01). Innovative behavior was positively correlated with psychological safety (*r* = 0.12, *p* < 0.05) and challenge-related stress (*r* = 0.17, *p* < 0.01). The correlation results provide a basis for testing our hypotheses.

**Table 2:** Means, standard deviations, and correlations.

Variables	Mean	*SD*	1	2	3	4	5	6	7	8	9
1 Education	3.78	0.82									
2 Gender	0.55	0.50	−0.06								
3 Age	31.82	6.69	0.07	0.04							
4 Marry	0.66	0.47	0.01	−0.07	0.46^∗∗∗^						
5 Tenure	9.19	6.80	−0.10	0.04	0.88^∗∗∗^	0.40^∗∗∗^					
6 Position	1.43	0.56	−0.04	−0.14^∗^	0.20^∗∗^	0.13^∗^	0.22^∗∗^				
7 Abusive supervision	1.92	1.01	−0.31^∗∗∗^	−0.01	−0.11	−0.13^∗^	−0.02	0.14^∗^			
8 Psychological safety	4.20	0.95	0.20^∗∗^	0.03	0.02	0.06	−0.03	−0.10	−0.41^∗∗∗^		
9 Challenge-related stress	2.65	0.90	−0.09	−0.15^∗^	−0.01	−0.06	0.01	0.14^∗^	0.22^∗∗^	−0.22^∗∗∗^	
10 Innovative behavior	4.19	0.86	−0.01	−0.01	0.12^∗^	0.05	0.09	0.16^∗∗^	−0.17^∗∗^	0.12^∗^	0.17^∗∗^

*N = 253, ^∗^p < 0.05, ^∗∗^p < 0.01, ^∗∗∗^p < 0.001.*

### Hypotheses Testing

We used Mplus to conduct the SEM and tested the entire model. In order to get the optimal model, we added the direct path from abusive supervision to innovative behavior based on the theoretical model. Results showed that both the theoretical model (χ^2^ = 550.63, df = 287, χ^2^/df = 1.92, RMSEA = 0.06, CFI = 0.94, NNFI = 0.93) and alternative model (χ^2^ = 543.34, df = 286, χ^2^/df = 1.90, RMSEA = 0.06, CFI = 0.94, NNFI = 0.93) fit the data well. We followed the suggestion of Little (1997) and found that NNFI almost did not change when the added path was included. According to the principle of model parsimony, the theoretical model was the most preferred model in our data analyses.

**FIGURE 2:**
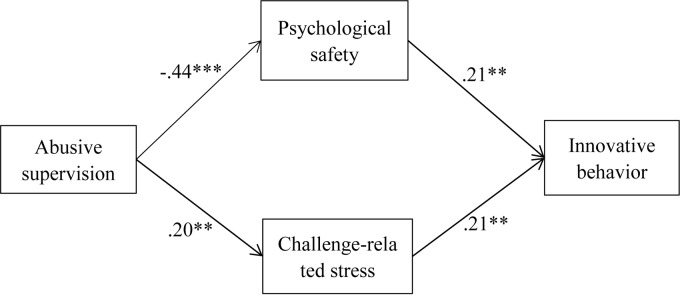
Results of theoretical model by using Mplus. *N* = 253, ^∗^*p* < 0.05, ^∗∗^*p* < 0.01, ^∗∗∗^*p* < 0.001. Standardized path coefficients are reported. Control variables are gender, age, marry, tenure, education, and position.

Figure 2 shows the standardized coefficients for the structural paths estimated in the theoretical model. Results showed that abusive supervision was negatively related to psychological safety (β = −0.44, *p* < 0.001) and that psychological safety was positively related to innovative behavior (β = 0.21, *p* < 0.01), after controlling gender, age, marry, tenure, education and position. The indirect effect from abusive supervision to innovative behavior through psychological safety was significant (indirect effect = −0.09, Bootstrap = 5000, 95% CI = −0.20, −0.01). Thus, Hypothesis 1 was supported.

Results also showed that abusive supervision was positively related to challenge-related stress (β = 0.20, *p* < 0.01) and that challenge-related stress was positively related to innovative behavior (β = 0.21, *p* < 0.01), after controlling gender, age, marry, tenure, education and position. The indirect effect from abusive supervision to innovative behavior through challenge-related stress was significant (indirect effect = 0.04, Bootstrap = 5000, 95% CI = 0.01, 0.09). Thus, Hypothesis 2 was supported.

### Robustness Test

We ran a robustness check to deepen our analysis and bolster our findings. Recently, Bayesian methods have been proposed as important complementary approaches for testing for mediation and computing the value of the mediated effect for its accuracy in small samples and providing the whole distribution not assumed to be normal (Miočević et al., 2017). In our robustness test, Bayesian analysis, because of its high statistics power (Miočević et al., 2017), was also employed to test our model. We use the key syntax of “estimator = bayes” in Mplus to run the Bayesian analysis with diffuse priors. It is important to consider convergence in Bayesian analysis carefully. The default convergence criterion is that a proportional scale reduction (PSR) factor is close enough to 1 for each parameter (Muthén, 2010). For this study, the largest PSR value at 10,000 iterations is 1.079. Results from Bayesian analysis using 10,000 iterations also showed that abusive supervision was negatively related to psychological safety (mean of β = −0.44, one-tailed *p* = 0.000, 95% CI = −0.56, −0.31) and that psychological safety was positively related to innovative behavior (mean of β = 0.19, one-tailed *p* = 0.003, 95% CI = 0.06, 0.33), after controlling gender, age, marry, tenure, education, and position. The indirect effect from abusive supervision to innovative behavior through psychological safety was significant (mean of indirect effect = −0.09, one-tailed *p* = 0.003, 95% CI = −0.16, −0.02). Thus Hypothesis 1 was also supported.

Results also showed that abusive supervision was positively related to challenge-related stress (mean of β = 0.20, one-tailed *p* = 0.002, 95% CI = 0.07, 0.33) and challenge-related stress was positively related to innovative behavior (mean of β = 0.21, one-tailed *p* = 0.002, 95% CI = 0.08, 0.33), after controlling gender, age, marry, tenure, education, and position. The indirect effect from abusive supervision to innovative behavior through challenge-related stress was significant (mean of indirect effect = 0.04, one-tailed *p* = 0.004, 95% CI = 0.01, 0.09). Thus, Hypothesis 2 was also supported.

## Discussion

The study builds on antecedent-benefit-cost framework and social cognitive theory to examine how abusive supervision impacts subordinates’ innovative behavior. Our findings showed that abusive supervision has an opposite directional influence on innovative behavior through two different mediating variables. Specifically, abusive supervision discouraged subordinates’ innovation through reducing subordinates’ psychological safety but promoted subordinates’ innovation through enhancing challenge-related stress. Our findings illustrated the complex mediating process of abusive supervision’s impact on innovative behavior, presenting a complementary explanation of the contradictory relationship between abusive supervision and innovative behavior. Also, we validated antecedent-benefit-cost framework and social cognitive theory, and shed light on two specific psychological cognitive mechanisms of social cognitive theory in the leadership field. Below, we discuss the theoretical and practical implications of our findings.

### Theoretical Implications

First, our research helps to resolve questions arising from theoretical and empirical researches, which suggest that abusive supervision and innovative behavior are sometimes negatively and sometimes positively correlated (Liu W. et al., 2012; Lee et al., 2013; Qin et al., 2017; Tepper and Park, 2017). Previous studies tried to explain conflicting findings from the perspective of context (Zhang and Liu, 2018). We introduced the antecedent-benefit-cost framework to the leadership field to presents an empirically supported complementary explanation of the contradictory relationship between abusive supervision and innovative behavior from the perspective of the mediating mechanism. Accordingly, our work verified the validity of the antecedent-benefit-cost framework.

Second, although the cost mediator variables via the process of abusive supervision negatively influencing innovative behavior have been frequently discussed in the literature (Liu D. et al., 2012; Liu W. et al., 2012), the beneficial mediator variables via its positive influence on innovative behavior are overlooked. Our approach takes a more united and integrative view to understand the complex mediating process of abusive supervision’s impact on innovative behavior. By integrating social cognitive theory and the results of previous studies, we explored the cost and benefit mediator variables in one model to demonstrate the underlying mechanisms of abusive supervision that has opposite directionalities influence on innovative behavior. Most importantly, we identified two specific psychological cognitive mechanisms of social cognitive theory in the leadership field, which verified and enriched social cognitive theory.

Finally, our work enriches the literature about abusive supervision. It is of vital importance to understand that the “positive effects” and “dark side” of abusive supervision coexist. The “dark side” of abusive supervision has been frequently discussed in the literature, nevertheless, most of the existing studies did not capture or reflect possible positive effects of abusive supervision, especially in the Asia-Pacific region with high power distance culture (Zhang and Liu, 2018). Our work showed that abusive supervision positively influences innovative behavior through challenge-related stress in China, which bridges this gap and echoes the call from Tepper and Park (2017) and Zhang and Liu (2018) to uncover the positive effects of abusive supervision.

### Practical Implications

In addition to the theoretical contributions of this work, our findings also provide guidance for practitioners. To begin, organizations should develop training programs for supervisors to train them to realize the “positive effects” and “dark side” of abusive supervision. Accordingly, organizations may need to take measures to reduce the negative effects of abusive supervision, such as providing a friendly working environment to facilitate subordinates’ psychological safety.

In addition, supervisors should pay attention to subordinates’ psychological safety and challenge-related stress. More importantly, when they want to activate subordinates’ challenge-related stress through criticism, supervisors should recognize that criticism may lead to psychological insecurity. Thus, after such criticism, supervisors should proactively care about subordinates through providing them with additional support and guidance. In doing so, supervisors could strengthen “positive effects” of abusive supervision and reduce the “dark side” of abusive supervision to better promote subordinates’ innovative behavior.

### Limitations and Future Research

First, we used a self-report questionnaire which can lead to common method bias (Podsakoff et al., 2003). Although the two-time point design could reduce common method bias and results showed that the common method bias had not seriously affected our results, it would be much better to use multi-sources data in future research. It is a better strategy to conduct three waves’ data collection for mediation analysis. Specifically, in future research projects, we suggest the subordinates complete questionnaires on abusive supervision at time point 1 and questionnaires on psychological safety and challenge-related stress at time point 2. The supervisor evaluates their subordinates’ innovative behavior at time point 3. In addition, the convenience samples may be not representative of the larger population in China and it is recommended to employ randomly selected samples in future research.

The current paper tested the self-defense mechanism and self-presentational mechanism via which abusive supervision has opposite directional influence on innovative behavior. We encourage researchers to examine other underlying mechanisms, such as emotional mechanism that may explain the possible positive effects of abusive supervision. In addition, Lin et al. (2016) found that ethical leader behaviors can come at some cost to the actor, thereby providing some clues for future research. Future research may explore the “dark side” of positive leadership, such as ethical leadership, and the underlying mechanism.

## Ethics Statement

In this study, data were collected by JZ at Minzu University of China. Institutions in China do not have Institutional Review Board. As a protection of all participants, all subjects read and sign an informed consent form before participating in this study and voluntarily made their decision to complete surveys. The protocol was approved by the Beijing Social Science Foundation and funded as a research project.

## Author Contributions

JZ developed the theoretical model and hypotheses, and wrote the manuscript. BZ provided comments on different versions of the manuscript and edited the manuscript.

## Conflict of Interest Statement

The authors declare that the research was conducted in the absence of any commercial or financial relationships that could be construed as a potential conflict of interest.
